# Functional Disruption of IQGAP1 by Truncated PALB2 in Two Cases of Breast Cancer: Implications for Proliferation and Invasion

**DOI:** 10.3390/biomedicines13081804

**Published:** 2025-07-23

**Authors:** Natalia-Dolores Pérez-Rodríguez, Rita Martín-Ramírez, Rebeca González-Fernández, María del Carmen Maeso, Julio Ávila, Pablo Martín-Vasallo

**Affiliations:** 1 Departamento de Oncología Médica, Hospital Universitario Nuestra Señora de la Candelaria, 38010 Santa Cruz de Tenerife, Spain; 2 Laboratorio de Biología del Desarrollo, UD de Bioquímica y Biología Molecular, Universidad de La Laguna, 38206 San Cristóbal de La Laguna, Spain; rmartira@ull.edu.es (R.M.-R.); refernan@ull.edu.es (R.G.-F.); javila@ull.edu.es (J.Á.); 3 Instituto de Tecnologías Biomédicas, Universidad de La Laguna, 38206 San Cristóbal de La Laguna, Spain; 4 Servicio de Anatomía Patológica, Hospital Universitario Nuestra Señora de Candelaria, 38010 Santa Cruz de Tenerife, Spain; mmaefor@gmail.com

**Keywords:** truncated PALB2, IQGAP1, invasive ductal carcinoma, cytoskeletal remodeling, breast cancer, cell migration, homologous recombination repair

## Abstract

**Background/Objectives:** Truncating mutations in *PALB2*, a critical component of the BRCA1-PALB2-BRCA2 homologous recombination repair complex, are associated with increased risk and aggressiveness of breast cancer. The consequences of PALB2 truncation on the expression, localization, and functional dynamics of the scaffold protein IQGAP1 were investigated in this study based on two cases of truncated PALB2 human breast invasive ductal carcinoma (IDC), specifically, c.1240C>T (p.Arg414*) and c.2257C>T (p.Arg753*). **Methods:** Using confocal microscopy, we examined co-expression patterns of IQGAP1 with PALB2, PCNA, CK7, and β-tubulin in tumor tissues from both control cancer and *PALB2*-mutated cases. **Results:** In PALB2-truncated tumors, IQGAP1 exhibited enhanced peripheral and plasma membrane localization with elevated co-localization levels compared to controls, suggesting altered cytoskeletal organization. PALB2 truncation increased nuclear and cytoplasmic N-terminal PALB2 immunoreactivity, indicating the presence of truncated isoforms disrupting the homologous recombination repair system. Co-expression analyses with PCNA revealed an inverse expression pattern between IQGAP1 and proliferation markers, suggesting S-phase cell cycle-dependent heterogeneity. Furthermore, the loss of IQGAP1 dominance over CK7 and β-tubulin in mutant tumors, along with persistent intercellular spacing, implied a loss of cell–cell cohesion and the acquisition of invasive traits. **Conclusions:** These data support a model where PALB2 truncation triggers a reorganization of IQGAP1 that disrupts its canonical structural functions and facilitates tumor progression via enhanced motility and impaired cell–cell interaction. IQGAP1 thus serves as both a functional effector and potential biomarker in *PALB2*-mutated IDC, opening novel paths for diagnosis and targeted therapeutic intervention.

## 1. Introduction

PALB2 (partner and localizer of BRCA2), also known as FANC-N for Fanconi anemia subtype N, has fundamental functions in dsDNA repair via homologous recombination (HR) in mammals [1,2] and serves as a scaffold protein [3,4] that links the whole BRCA complex. Truncated monoallelic or heterozygous alterations increase the risk of breast, ovarian, and pancreatic cancer; in the case of breast cancer, the risk is 5 to 7 times higher [5].

**Figure 1:**
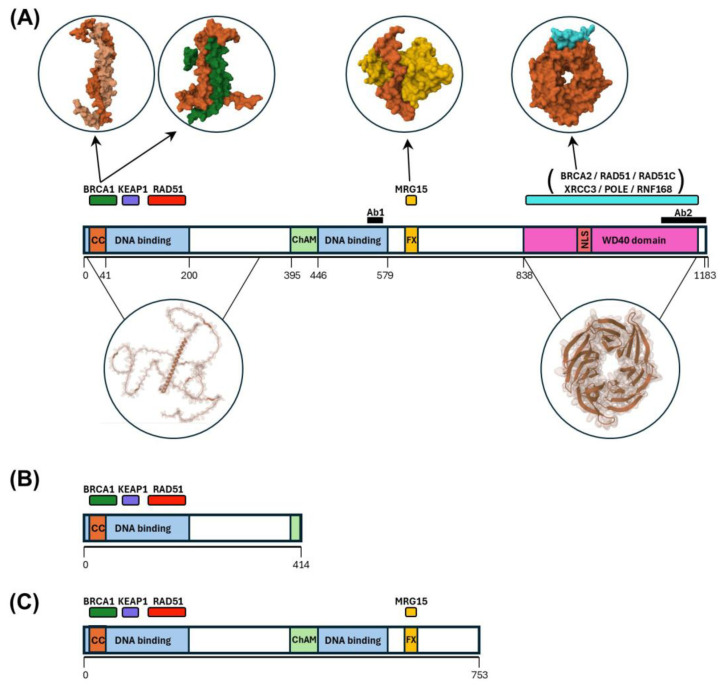
Key domains and interaction regions of PALB2 protein and the P-T1 and P-T2 variants. (**A**) Domains and interaction regions of the PALB2 protein. The upper circles show the molecular surface representation of the Coiled–coil PALB2 (orange) dimerization, coil–coil PALB2-BRCA1 (green) interaction, PALB2-MRG15 (yellow) interaction, and PALB2-BRCA2 (cyan) interaction. The lower circles show the intrinsically disordered structure of the N-terminal (AlphaFold 2 prediction) and the β-sheet structure of the WD40 domain at the C-terminal. Ab1 and Ab2 represent the antigenic regions recognized by the PA5-48258 and BS-0588R PALB2 antibodies, respectively. (**B**,**C**) Diagram of the domains and interaction regions of PALB2 variants P-T1 and P-T2, respectively. CC, coil–coil motif; ChAM, chromatin association motif; FX, FXLP motif; NLS, nuclear export signal; BRCA1, BRCA1 DNA repair-associated; KEAP1, Kelch-like ECH-associated protein 1; RAD51, RAD51 recombinase; MRG15, MORF-related gene 15; BRCA2, BRCA2 DNA repair-associated; RAD51C, RAD51 paralog C; XRCC3, X-ray repair cross complementing 3; POLE, DNA polymerase epsilon (catalytic subunit) and RNF168, ring finger protein 168.

The human PALB2 protein comprises 1186 amino acids and spans specific binding domains from the N to C termini for BRCA1, KEAP1, RAD51, DNA, chromatin binding (ChAM), MRG15, RAD51, RAD51C, XRCC3, BRCA2, and Pol η (Figure 1A). PALB2 possesses an N-terminal coil–coil domain that negatively regulates HR via PALB2 oligomerization [6,7] and a WD40-type β-propeller with seven blades, which is the binding site for the N-terminus of BRCA2 [8]. Furthermore, the function of PALB2 is regulated by phosphorylation [9,10], ubiquitylation [11], and interaction with RNF168 (E3 ubiquitin ligase) [12].

IQGAP1 (IQ motif containing GTPase activating protein 1) is a remarkably flexible scaffolding multidomain protein that regulates complex cellular processes and signaling pathways, such as cell fate, cell migration, cell cycle, angiogenesis, and many others, most of them being involved in proliferation, tumorigenesis, cancer, and cell migration [13,14]. More complete information regarding protein structure, functional domains, and other properties can be found in references [15,16,17].

IQGAP1 has been shown to be a partner of BRCA1 that influences mislocalization and imbalanced subcellular distribution of both BRCA1 and IQGAP1 associated with centrosome anomalies. BRCA1 inhibits centrosome amplification by controlling the stability of centrosome proteins; in contrast, BRCA1 depletion leads to supernumerary centrosomes [18,19,20,21,22].

Given the molecular-functional association of IQGAP1 and BRCA1 and BRCA1 with PALB2 [22], our purpose was to explore the subcellular location and expression levels of IQGAP1 and its relationship with PALB2 in breast invasive ductal carcinoma (IDC) samples from patients with PALB2 functional deficiency because of pathogenic variants in the germ line. For Western blotting, PCR, and other broadly used molecular techniques, severance and homogenization of the sample are mandatory for studying protein or gene expression. These techniques do not provide detailed information about the involvement of different cell types or subcellular compartments but, instead, a general idea of what occurs in a biologically heterogeneous sample. To gain a better insight, we performed co-immunolabeling with several markers and proteins analyzed using confocal microscopy with the aim of obtaining high-quality cell-by-cell and subcellular data in human breast cancer samples.

This study demonstrates that truncating mutations in the *PALB2* gene result in marked alterations in the expression and subcellular localization of both PALB2 protein variants and IQGAP1 in IDC. Our data also suggest the role of IQGAP1 in modulating proliferative dynamics, possibly indicating distinct subpopulations within the same cell cycle phase in these tumor cells.

## 2. Materials and Methods

### 2.1. Ethical Considerations

This study was approved by the Ethics Committee of La Laguna University, Canary Islands, Spain, and the Ethics Committee of the Hospital Universitario Nuestra Señora de Candelaria (HUNSC) CHUNSC-2022-116, 11 November 2022. We analyzed two wild PALB2-gene breast cancers as controls and two different truncated PALB2-gene breast carcinomas. The subjects signed an informed consent document before participating in the project.

### 2.2. Patients with Truncated PALB2 Breast Carcinomas and Breast Cancer Controls

PALB2-truncated variant case 1 (P-T1): A 45-year-old patient diagnosed with metastatic breast carcinoma with liver involvement; ER-, PR-, and human epidermal growth factor receptor 2-negative (HER2-); Ki 67 15%; programmed cell death ligand 1 (PD-L1)-positive; triple-negative subtype. The entire coding sequence was analyzed, and exon–intron flanking regions of the PALB2 gene were determined using a capture kit biotinylated probe. This was followed by ultrasequencing on an Illumina MiSeq system and the subsequent analysis of SNVs, indels, CNVs, and ALU insertions with the Datagenomics^®^ platform, showing a c.1240C>T (p.Arg414*) (Figure 1B) variant, classified as a pathogenic variant in ClinVar. This sequence change creates a premature stop signal (p.Arg414*) in the PALB2 gene. This variant is present in the GnomAD control population database at a very low frequency (0.00001) and has been reported in individuals affected with breast or ovarian cancer (PMID: 21285249, 21165770, 21618343, 22692731, 24136930, 24448499). Loss-of-function variants in PALB2 are known to be pathogenic (PMID: 17200668, 24136930, 25099575).

PALB2-truncated variant case 2 (P-T2): A 40-year-old patient diagnosed with IDC (IDC) of the left breast G2 pT1 N1mic M0, estrogen receptors (ERs) 75% immunostained tumor nuclei, progesterone receptors (PRs) 84% immunostained tumor nuclei, Ki.67 36.97% of immunoreactive tumor nuclei and HER2-negative, luminal B subtype. The patient’s mother had been diagnosed with breast and ovarian carcinoma at the age of 27 years old and died when she was 29 years old. From a total blood sample, DNA was isolated and quantitative and qualitative assessment of the DNA sample conducted. Capture and enrichment of the exonic regions and the flanking intronic zones of the genes contained in the TruSight™ Cancer sequencing panel (Illumina, Albany, NY, USA) were performed with Nextera Rapid Capture™ technology and massive sequencing with the NextSeq™ sequencer (Illumina) showing a c.2257C>T (p.Arg753*) (Figure 1C) variant in the PALB2 gene. This mutation is a missense variant that results in the substitution of an arginine amino acid for a premature stop codon at position 753 of the protein. It is described in the HGMD clinical databases (CM070237) as a pathogenic variant associated with Fanconi anemia and in the ClinVar database (142403) as a pathogenic variant associated with Cancer Predisposition Syndrome and Familial Breast Cancer.

Breast cancer control case 1 (CC1): A 61-year-old woman, diagnosed in November 2020 with IDC Nottingham grade 1 of the left breast pT1cpN0. ER 96.5%, PR 28.9%. Ki-67 of 16.3%. HER2-, CK19 diffusely positive. Luminal B. Negative genetic study. Nottingham grade 1, pathology stage: ductal carcinoma in situ, pT1c.

Breast cancer control case 2 (CC2): A 57-year-old woman, diagnosed with invasive ductal breast carcinoma (IDC) in June 2022, G1, pT2 pN1mi ICD, ER 87%, PR 91%, Ki-67 of 22%, HER2-. Genetic study: negative. Nottingham grade 1, associated with extensive peripheral ductal carcinoma in situ. Pathology stage (AJCC/UICC 8th ed.): pT2 pN1mi(sn). Quantification was performed using the Aperio ImageScope 12.4.6 Analysis System Software (Leica Biosystems, Buffalo Grove, IL, USA): HER2-. Quantification was conducted according to ASCO/CAP Guidelines for HER2 evaluation update of May 2018.

None of the cases underwent any oncological or medical treatment prior to surgery for tumor resection.

### 2.3. White Cell Purification and Protein Detection

Circulating white cells were isolated from whole blood samples via density-gradient centrifugation using Histopaque-1077 (Cat. #10771, Sigma-Aldrich, Irvine, UK). After isolation, cells were counted, aliquoted at 100,000 cells each, and stored at −80 °C. Cell samples were incubated with LBS for 10 min at 95 °C for Western blotting. Whole cell lysates were electrophoresed on a denaturing 4–20% polyacrylamide gel and transferred to Immobilon™-P membranes (Millipore, Bedford, MA, USA) via electroblotting. Membranes were blocked in PBS/5% BSA for 1 h. Protein detection was performed using PALB2 (C-terminus) antibody (1:1000, Cat.# BS-0588R, Bioss, Woburn, MA, USA) and anti-rabbit Ig horseradish peroxidase-conjugated secondary antibody (Cat. # NA9340, Cytiva, Marlborough, MA, USA). Detection was performed on a ChemiDoc XRS (Bio-Rad Laboratories, Hércules, CA, USA) using Immobilon Western Chemiluminescent HRP substrate (Millipore, Bedford, MA, USA) reagents following the manufacturer’s instructions. Protein signal intensities were normalized against the total protein level in each lane, quantified via Coomassie Brilliant Blue staining, and analyzed using Image Lab 6.1 software (Bio-Rad Laboratories, Hércules, CA, USA).

### 2.4. Immunohistochemistry

Paraffin-embedded tissue sections were cut into five-micron-thick segments. Tissue sections were deparaffinized in xylene and rehydrated in a graded series of alcohol baths. Epitope retrieval was performed in an autoclave, heating samples at 120 °C for 10 min in sodium citrate buffer (pH 6.0). Antibodies used for this study are specified in Table 1. Antibody against PALB2 N-terminal domain antibody a was used to label both proteins in full-length and truncated form, and antibody against PALB2 C-terminal domain bound only to the complete protein (wild type) and that of case P-T2. After non-specific sites had been blocked with 5% bovine serum albumin in Tris-buffered saline (TBS) for 1 h at room temperature, tissue sections were incubated overnight at 4 °C simultaneously with a mixture of distinct primary antibodies (a complete list of antibodies is shown in Table 1).

Samples incubated without primary antibodies were used as a negative control. Slides were washed three times with TBS and then incubated at room temperature in dark for 1 h with a mixture of two or three secondary antibodies: FITC conjugated against rabbit [Cat #F9887, Sigma-Aldrich, Irvine, UK] and DyLight^®^ 650-conjugated against mouse [Cat # ab97018, Abcam, Cambridge, UK]. Finally, slides were mounted with ProLong^®^ Diamond Anti-Fade Mountant with DAPI (Molecular Probes by Life Technologies, Eugene, OR, USA) and analyzed using a Leica SP8 confocal microscope (Leica Microsystems, Wetzlar, Germany). Digital images were obtained in the raw Leica Image Format (LIF) and exported as Joint Photographic Experts Group (JPEG) files at 300 ppi. Figures were assembled using Microsoft PowerPoint 2019 and exported at 300 ppi.

### 2.5. Qualitative and Semi-Quantitative Image Analysis

Although we only present the results of the quantitative analysis in this report, being aware of the possible errors in the use of plugins [23], we performed a blind qualitative and semi-quantitative analysis, which we then compared with the quantitative analysis specified below in the quantitative analysis section. We did so as follows: two independent observers evaluated preparations and photographs blindly, grading the staining intensities as absent (−), faint (+), moderate (++), or strong (+++). Cutoffs were established through consensus among observers after an initial review of several samples of varying appearance and blind coding. In cases where the score data differed by more than one unit, the means of the score data were calculated.

### 2.6. Quantitative Image Analysis

ImageJ 1.54g software (National Institutes of Health; Bethesda, MD, USA) was used for image analysis with the EzColocalization plugin [24]. Confocal photographs used were taken using the same parameters. Laser power and detector gain settings were set to cover optimized fluorescence signals in a 16-bit depth range without saturation. Fluorescence changes from baseline were measured as the mean intensity of selected regions of interest, and 60 measurements (at least) were performed per photograph in 6 photographs (at least) of different fields for each different immunostaining process. In addition, the “Cell Counter” plugin was used to ensure that cancer cells were counted only once. The Pearson correlation coefficient (PCC) was used to quantify the degree of colocalization between fluorophores [23,25].

### 2.7. Statistics

Statistical analysis was carried out using SPSS version 29.0 for Windows (IBM Corp., Armonk, NY, USA). For panels A and B in Figure 7, the immunofluorescence intensity was quantified for each specific immunohistochemical marker corresponding to the analyzed proteins. The non-normal distribution of the data was confirmed using the Kolmogorov–Smirnov test. Differences in staining intensity between the control and specific PALB2 isoforms were assessed using the non-parametric Kruskal–Wallis test [26]. A *p*-value of < 0.05 was considered statistically significant. To determine the significance of the slopes in Figure 7, panels C and D, quantitative variables were summarized as the mean ± s.e.m. The equality of variances was tested with Levene’s test. Normally distributed continuous variables with equal variances were analyzed via analysis of variance, and Student’s *t*-test was used to test the difference between the response of two groups (IQGAP1-PALB2, -PCNA, -CK7, and -β-tubulin); as a means test of the points obtained, if variances were not equal, the Kruskal–Wallis test was performed. *p* > 0.05 was considered statistically significant.

## 3. Results

### 3.1. PALB2 Western Blot from Circulating White Cells

**Figure 2:**
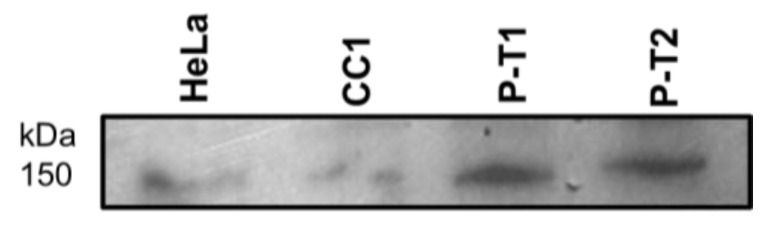
PALB2 full-length protein. Western blot of isolated proteins from HeLa cells and from circulating white cells from control IDC and cases with truncated variants of PALB2, P-T1, and P-T2 probed with PALB2 C-terminus antibody.

Figure 2 shows a Western blot of proteins isolated from circulating white cells from control 110981 and cases with truncated variants of PALB2, P-T1, and P-T2 and proteins from HeLa cells probed with antibody against the C-terminus of PALB2. All samples, at different intensities, express the PALB2 full-length protein.

### 3.2. PALB2 and IQGAP1 Co-Expression in Breast IDC

**Figure 3:**
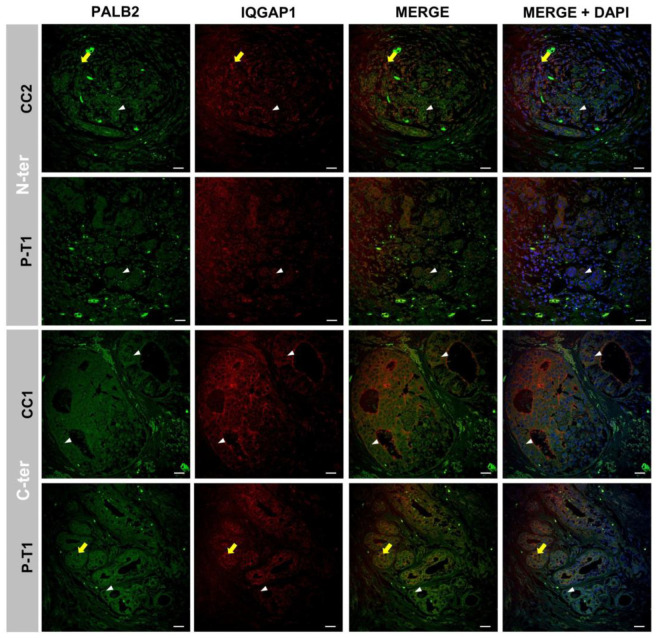
PALB2 (green) and IQGAP1 (red) expression in breast cancer control (CC1 and in CC2) mutated breast cancer (P-T1) labeled with antibodies binding to N-terminal or C-terminal domains. In CC2, PALB2-specific fluorescence at high intensity in cytosol and to a lesser extent in the nuclei of tumor cells (white arrowheads); IQGAP1-specific immunofluorescence in the plasma membrane and cytosol of most cells (yellow arrows) at two different intensities, high and medium level, in a dot-like pattern distribution; in the PALB2-IQGAP1 merged image, these two levels are more evident, and colocalization exists to a certain extent. B-T1 anti-PALB2-N-terminus antibody immunofluorescence signal present in both cytosol (high) and nucleus (medium and homogeneous); IQGAP1-specific immunofluorescence is displayed at a homogeneous medium intensity level in the plasma and cytosol of most cells. C-terminus anti-PALB2 antibody in CC1 samples exhibited similar immunolabeling to the N-terminus (white arrowheads). The IQGAP1 localization pattern of two intensity levels and localization is also shown in the first lane of the images. B-T1 and C-terminus anti-PALB2 antibody fluorescence at medium–high intensity, mainly in cytosol and at much lower intensity in the nuclei of cancer cells (white arrowheads). IQGAP-specific immunofluorescence at medium- to high-level intensity in cytosol and plasma membrane, frequently colocalizing (merged) in some tumor areas (yellow arrows). Scale bar = 40 µm.

Figure 3 and Figure S1 display representative images of PALB2 and IQGAP1 immunolocalization in breast cancer control references (cases CC2 and CC1) and in *PALB2*-mutated breast cancer (case P-T1, c.1240C>T; p.Arg414*) labeled with antibodies binding to N-terminal or C-terminal domains. In the control case, PALB2 exhibited specific fluorescence at high intensity in cytosol and to a lesser extent in the nuclei of tumor cells.

Tumoral cells were evaluated using ImageJ in most of the confocal microscopy photographs taken; however, we selected those considered better to illustrate the quantitative data. IQGAP1-specific immunofluorescence is found in the peri-plasma membrane zone and cytosol of most cells at two different intensities—namely, high and medium levels—in a dot-like pattern distribution. In the PALB2-IQGAP1 merge image, these two intensity levels were more evident and revealed partial colocalization (Pearson’s correlation coefficient PCC = 0.47, *p* < 0.05; Figure 7E). In *PALB2*-mutated case P-T1, the anti-PALB2-N-terminus antibody immunofluorescence labeling signal was present at a high intensity level in cytosol and at medium and homogeneous intensity in the nuclei of tumor cells. IQGAP1-specific immunofluorescence was displayed at a homogeneous medium intensity level within the plasma membrane and bordering the plasma membrane and cytosol of most cells (Figure 3 and Figure S1).

C-terminus anti-PALB2 antibody in samples from control CC1 exhibited similar immunolabeling to the N-terminus. The IQGAP1 localization pattern displayed the two intensity levels and localization shown with the N-terminal antibody in control CC2 images (first lane in Figure 3). Samples from the mutated *PALB2*-mutated case (deletion) probed with C-terminus anti-PALB2 antibody presented fluorescence at medium–high intensity levels mainly in cytosol and at much lower intensity in the nuclei of cancer cells, as shown in the lower lanes in Figure 3 and Figure S1. IQGAP-specific immunofluorescence at medium- to high-level intensity is presented in cytosol and plasma membrane, frequently colocalizing with PALB2 (merging) in some tumor areas (PCC = 0.67 *p* < 0.05; Figure 7E).

### 3.3. IQGAP1 Expression and Cellular Proliferation in Tumor Breast with Truncated PALB2 Scaffold Protein

**Figure 4:**
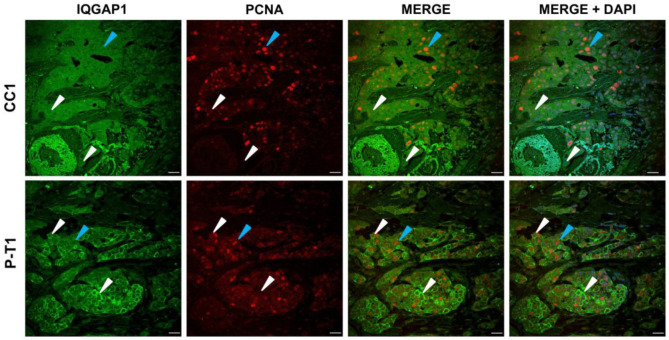
IQGAP1 (green) expression and cell proliferation. Control: IQGAP1-specific immunofluorescence at medium intensity level and PCNA (red) at heterogeneous, low (white arrowheads) to high (blue arrowheads). P-T1 case: staining for IQGAP1 at medium to high intensity levels and PCNA low (white arrowheads) to high (blue arrowheads). Scale bar = 40 µm.

Co-expression of IQGAP1 and PCNA, the DNA clamp processivity factor of DNA polymerase δ in eukaryotic cells, was analyzed in order to evaluate the involvement of IQGAP1 expression in cell proliferation. In samples from control CC1, IQGAP1-specific immunofluorescence is found at a medium intensity level and PCNA at heterogeneous, low to high intensity, as shown in Figure 4 (upper lane). The *PALB2*-mutated case P-T1 fluorescence signal for IQGAP1 was present at medium to high intensity levels and at variable intensities for PCNA from zero to high, and was less abundant than in the control specimen.

Cells in control tumor samples had PCNA+ staining of 67 ± 10.1% at variable intensity. In the truncated PALB2 case P-T1, 76.2 ± 10.0% (*p* > 0.05) of cells were PCNA+. Correlation between IQGAP1 and PCNA expression levels evidenced that, in 27 ± 5.4% of cells, high expression of PCNA corresponded to lower expression of IQGAP1 and vice versa, indicating an inverse relationship. This fact reached a higher value in samples from *PALB2*-gene-mutated ductal breast (30 ± 4.1%, *p* > 0.05; Figure 7C).

### 3.4. IQGAP1 Involvement in Cellular Migration and Invasion in IDC with Truncated PALB2

**Figure 5:**
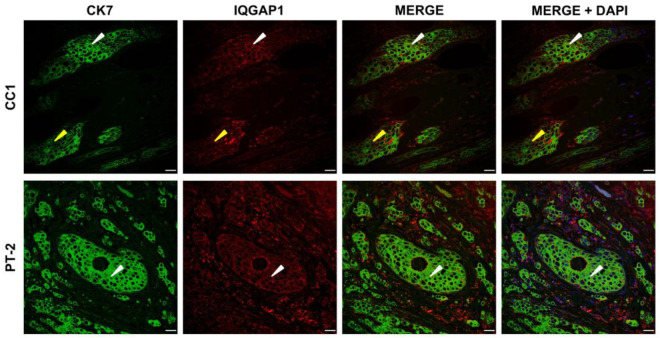
IQGAP1 (red) and CK7 (green) co-staining. CC1, IQGAP1 immunofluorescence at medium intensity level in the periphery of cytosol and plasma membrane of tumor cells (white arrowheads) with no interphase between cells and CK7 (green) at heterogeneous, high to saturated intensity in the cytosol of tumor cells and evident interphase space between cells. P-T2, staining for IQGAP1, medium intensity in cytosol, becoming high-intensity in the periphery and in the plasma membrane. CK7 staining is at saturation level in the cytosol of tumor cells, and intercell space is evident. In the merging of some tumor cells of the control case, the expression intensity of IQGAP1 (yellow arrowheads) is prevalent over CK7 and never in the P-T case. Scale bar = 40 µm.

IQGAP1 and intermediate filament CK7 [27] and IQGAP1-β-tubulin [15,28] co-labeling was accomplished to gain insight into possible changes elicited by truncated PALB2 in the role of IQGAP1 in cell adherence, cell–cell interaction, and movement via cytoskeletal reorganization. Representative images of IQGAP1 and CK7 co-staining are shown in Figure 5. In control samples, IQGAP1 displayed clear specific immunofluorescence at a medium intensity level in the periphery of cytosol and plasma membrane of tumor cells (white arrowheads) with no interphase between cells and the CK7 fluorescence signal at heterogeneous, high to saturated intensity in the cytosol of tumor cells with evident interphase space between cells. Samples from the *PALB2*-mutated case P-T2 showed staining for IQGAP1 at medium intensity in cytosol, becoming high-intensity in the periphery and in the plasma membrane; CK7-specific staining was at saturation level in the cytosol of tumor cells, leaving a noticeable intercell space. Interestingly, as seen in the merging, in some tumor cells of the control case, the expression intensity of IQGAP1 (yellow arrowheads) is prevalent over CK7 (15 ± 3.0%) and never in the truncated PALB2 case (Figure 7D).

**Figure 6:**
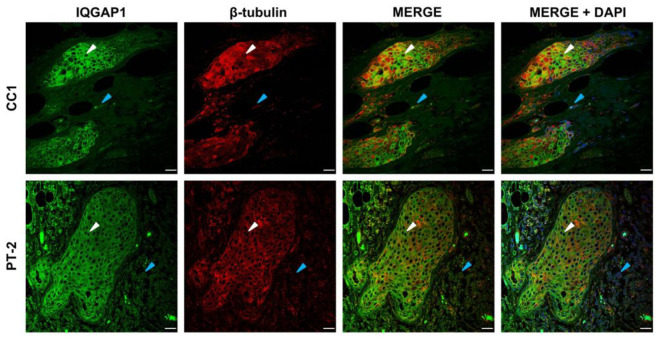
IQGAP1 (green) and β-tubulin (red) co-staining. CC1, IQGAP1, immunofluorescence at variable medium to high intensity levels in the periphery of cytosol and plasma membrane of tumor cells (white arrowheads) and at a lower intensity level in stroma cells, and β-tubulin (green) at heterogeneous, high to saturated intensity in the cytosol of tumor cells, myoepithelial cell layer (blue arrowheads) on the outside and a second focus of ductal carcinoma “in situ”. P-T2, IQGAP1 at medium intensity in cytosol and high intensity in the periphery and in the plasma membrane; β-tubulin immunofluorescence at a high intensity level in the cytosol of tumor cells and to a lesser extent in stroma cells (blue arrowheads). Scale bar = 40 µm.

IQGAP1 and β-tubulin co-staining is portrayed in Figure 6. IQGAP1 in control samples depicts the typical clear immunofluorescence signal at variable medium to high intensity levels in the periphery of cytosol and plasma membrane of tumor cells and at a lower intensity level in stroma cells. β-tubulin labeling is at heterogeneous, high to saturated intensity in the cytosol of tumor cells; in addition, in the portrayed sample, a myoepithelial cell layer on the outside is observed. IQGAP1 staining in case P-T2 reaches a medium intensity level in cytosol and high intensity in the periphery and in the plasma membrane; β-tubulin immunofluorescence has a high intensity level in the cytosol of tumor cells and to a lesser extent in stroma cells. Interestingly, merge images in control cases show some tumor cells in which the expression intensity of IQGAP1 (white arrowheads) is prevalent over β-tubulin, showing two well differentiated populations. In *PALB2*-mutated samples, there is a more homogeneous cell population in which IQGAP1 expression is prevalent and colocalization with β-tubulin is high. Regarding IQGAP1-β-tubulin in *PALB2*-mutated samples, there is a more homogeneous cell population in which IQGAP1 expression is prevalent and colocalization with β-tubulin is high, as shown in Figure 6.

**Figure 7:**
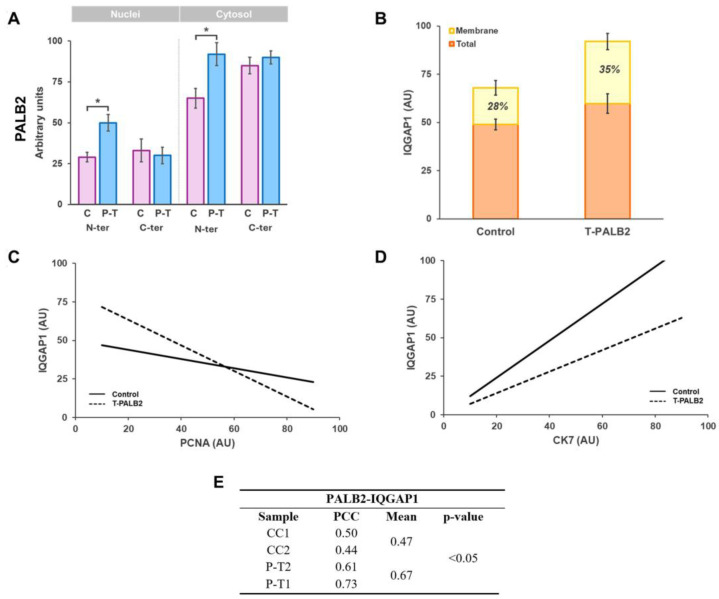
Diagrams of PALB2 and IQGAP1 expression in truncated PALB2 human breast invasive ductal carcinoma. AU—arbitrary units. (**A**) Specific PALB2 expression in nucleus or cytosol in control (CC) IDC and in truncated PALB2 (P-T) IDC samples probed with antibodies against N- or C-terminus of PALB2 protein (* *p* < 0.05). (**B**) IQGAP1 total expression and peri-plasma membrane expression (*p* < 0.05). (**C**) In subpopulations of cells (25–30% of total cells), correlation between the expression of IQGAP1 and PCNA evidenced that high expression of PCNA corresponded to lower expression of IQGAP1, which was higher in truncated PALB2 cases (*p* < 0.05). (**D**) Correlation of IQGAP1/CK7 expression-level quotients in control and in PALB2-truncated cases. The correlation is lower in PALB2-truncated cases (*p* > 0.05). In (**C**,**D**), point clouds are not represented to improve the clarity of the plots. (**E**) The Pearson correlation coefficient (PCC) was calculated for each sample to quantify the degree of colocalization between fluorophores.

Figure 7 summarizes the results reported in this study.

## 4. Discussion

This study provides novel insights into the subcellular distribution and potential interactions between PALB2 and IQGAP1 in human invasive ductal breast carcinoma, with a particular focus on the consequences of truncating PALB2 mutations. Our findings suggest a functional link between PALB2 protein and alterations in IQGAP1 expression and subcellular localization which may contribute to tumor progression through the deregulation of cellular proliferation, migration, and cytoskeletal organization. IQGAP1 was specifically upregulated in truncated PALB2 breast IDC compared with the corresponding adjacent noncancerous tissue, similar to results reported for human breast cancer tissues and cell lines [16]. PALB2 and IQGAP1 levels are higher in the cytosol of PALB2-mutated cases, with higher PCC (mean 0.47 vs. 0.67), particularly in the peripheral areas in the vicinity of the plasma membrane.

Semi-quantitative analyses coincided with those performed with ImageJ with the corresponding plugins, and the data are not included in this report.

### 4.1. Expression of PALB2 in Blood White Cells

Western blot analysis of PALB2 in circulating white blood cells revealed the detectable full-length protein in both controls and patients carrying truncating PALB2 variants (P-T1 and P-T2), albeit with variable intensities. While these findings confirm PALB2 expression in peripheral cells, the results may not accurately reflect the molecular consequences in breast epithelial tissue, where the pathogenic phenotype manifests. Therefore, caution is warranted when extrapolating these observations to breast cancer pathology. Further studies in breast-derived cells or tissues are needed to elucidate the functional impact of these variants. Nonetheless, alterations in peripheral white blood cells may hold predictive or surrogate value, potentially reflecting systemic responses to germline PALB2 mutations, and could aid as peripheral biomarkers in early risk stratification or monitoring in mutation carriers.

### 4.2. Expression and Co-Localization of IQGAP1 and PALB2 Protein Variants in Breast Cancer Control and in PALB2-Truncated Breast Cancer as Revealed by Antibodies Directed at the N-Terminus or the C-Terminus

Quantitative analysis of confocal microscopy images revealed distinct patterns of IQGAP1 and PALB2 expression across the two different ductal breast carcinomas after probing with anti-IQGAP1 antibody and anti-C-terminus PALB2 Ab; both cases reported in this study lack the BRCA2-interacting domain. Representative images are shown in C-term lines in Figure 3 and Figure S1.

In control samples, fluorescence intensity exclusive for IQGAP1 exhibited a relative intensity of 12.5 ± 2.5% in control tumor cells, 6.5 ± 1.5% specific for PALB2 alone, and co-localization of both markers was observed in only 5.0 ± 1.0% of signals. Conversely, case P-T1 demonstrated a markedly higher prevalence of expression, with 37.5 ± 2.5% of cells positive for IQGAP1, 22.5 ± 2.5% for PALB2, and 23.5 ± 3.6% co-expressing both proteins. These findings suggest an upregulation in the expression of both proteins in the PALB2-truncated case and potential interaction between IQGAP1 and PALB2 probably through BRCA1 [8] in a wide subset of tumor cells, highlighting tumoral heterogeneity.

Complementarily, our data suggests greater co-expression and colocalization of IQGAP1/PALB2 in samples from case P-T1 probed with C-terminus antibody compared to samples from the same tumor probed with the N-terminus antibody, as seen in the representative lines of the N-term and C-term of case P-T1 in Figure 3 and Figure S1. These results might be explained by the constant IQGAP1 expression in both cases and an increase in the PALB2 signal in case P-T1 when probed with the N-terminus antibody that is able to label not only the full PALB2 protein but also the truncated one.

Immunofluorescence analysis revealed a robust expression of PALB2 in both the cytoplasm and nuclei of tumor cells from control cases, in line with its known role in DNA repair through homologous recombination [29,30]. In PALB2-mutated tumors, N-terminal-specific antibodies detected intense cytoplasmic and moderate nuclear signals, suggesting the presence of truncated forms lacking the C-terminal region necessary for BRCA2 binding [8]. PALB2 thus seems to exist in two different molecular subpopulations, one containing the complete PALB2 form and a second with the truncated non-scaffolding form and, consequently, the incomplete non-canonical form in the cell nucleus could compete with the full BRCA1-PALB2-BRCA2 complex, impairing its performance within the homologous recombination repairing system.

### 4.3. IQGAP1 Expression Varies During the Cell Cycle

Figure 4 exhibits representative images for the expression and localization of IQGAP1 and PCNA proteins. Tumoral cells showed expression levels of IQGAP1 similar to those reported in the previous section. PCNA in the tumor control evidenced 67 ± 10.1% of cells in the S phase of cell cycle at variable fluorescence intensity as indicative of asynchrony. In the truncated PALB2 case P-T1, 76.2 ± 10.0% of cells were in the S phase.

Correlation between the expression of IQGAP1 and PCNA evidenced that in 27 ± 5.4% of cells, high expression of PCNA corresponded to lower expression of IQGAP1 and vice versa, that is, an inverse relationship (Figure 4 and Figure 7C). This fact seems to be more evident in the PALB2-gene-mutated ductal breast (30 ± 4%). However, when all data points are considered, statistical analysis pointed to no significant correlation (*p* < 0.05). This fact may indicate at least two different populations of cells, even if they are within the same stage of the cell cycle.

### 4.4. Truncated PALB2 Elicits Changes in the Involvement of IQGAP1 in Invasion and Migration in Invasive Ductal Carcinoma

Through double immunofluorescence labeling of IQGAP1 with CK7 and β-tubulin, we observed distinct subcellular localization patterns and co-expression dynamics that appear to be significantly influenced by the PALB2 mutation status.

In control IDC samples, IQGAP1 localized predominantly at the periphery of the cytosol and plasma membrane, with a distribution consistent with its known function in facilitating cell–cell contact and cortical actin organization [15]. CK7 signal intensity was strong and heterogeneous in cytosol, with clear intercellular boundaries, suggesting a well-maintained cellular architecture. Notably, a subset of tumor cells in control cancer samples displayed a higher IQGAP1 signal relative to CK7, hinting at a subpopulation with enhanced migratory or adhesive properties. In contrast, IDC samples harboring a truncated PALB2 (case P-T2) exhibited a shift toward higher peripheral and membrane-associated IQGAP1 expression, along with saturated CK7 staining and disrupted intercellular spacing. This phenotype may reflect a transition toward a more invasive cellular state. The absence of tumor cells with predominant IQGAP1 expression over CK7 in the mutant background further underscores a possible deregulation of IQGAP1 compartmentalization and function upon PALB2 truncation.

Co-staining with β-tubulin further revealed differential cytoskeletal organization between genotypes. In control samples, β-tubulin was heterogeneously distributed across the cytosol, and IQGAP1 expression identified distinct subpopulations of tumor cells, some of which displayed the enhancement of IQGAP1 over β-tubulin, suggesting functional compartmentalization and variable migratory potential [31]. Remarkably, PALB2 mutant samples displayed a more homogeneous tumor cell population with strong peripheral IQGAP1 expression and high colocalization with β-tubulin. This suggests a reinforcement of cytoskeletal dynamics conducive to coordinated migration or invasion [31].

IQGAP1 interaction with microtubules via β-tubulin and with intermediate filaments such as CK7 may serve to orchestrate changes in cellular motility by forming podosomes/invadosomes in a number of tumor cells [14], as our results indicate, via the presence of truncated PALB2, which can lead to changes in the spatial distribution and function of IQGAP1 within tumor cells, ultimately leading to extravasation and metastasis [16,18].

### 4.5. Discussion of the Study as a Whole

Mechanistically, PALB2 plays a pivotal role in homologous recombination and genome stability, but emerging evidence suggests that it may also regulate cytoskeletal integrity through DNA damage response crosstalk [22]. In addition to altered IQGAP1 distribution and colocalization with CK7 and β-tubulin—supporting a role in invasive potential—co-expression analysis with PCNA reveals a complex interplay between proliferation and structural dynamics. While overall PCNA+ cell percentages did not differ significantly between control and PALB2 mutant samples, an inverse correlation between PCNA and IQGAP1 expression was more pronounced in the truncated PALB2 case. This inverse pattern suggests that IQGAP1 may be differentially regulated in proliferating versus migrating cells, consistent with a phenotypic switch. Together, these data support a model in which PALB2 truncation contributes to IDC progression through pleiotropic modulation of both DNA replication machinery and cytoskeletal remodeling via IQGAP1.

The increased peripheral localization and higher intensity of IQGAP1 in PALB2-truncated samples support a model wherein IQGAP1 is recruited or retained at the cell membrane in response to cellular stress or dysfunction elicited by the compromised BRCA1-PALB2-BRCA2 axis. As PALB2 truncation impairs homologous recombination repair and promotes genomic instability, downstream effects on cytoskeletal regulation may enhance the motile and invasive phenotype of tumor cells through some mechanisms other than IQGAP1. Furthermore, the consistent intercellular space observed in both CK7- and β-tubulin-stained regions in PALB2 mutant tumors may suggest a loss of intercellular cohesion enhancing migratory and invasive potential, hallmark features of aggressive IDC phenotypes.

The limitations of this study are those inherent to studies in humans: The homogeneity of the samples means that it is difficult to find patients with the same or similar mutations or proteins. In addition to the heterogeneity of the cases, we must mention the cellular heterogeneity of the samples. Consequently, we must be cautious in generalizing the described phenomenon of molecular interaction between PALB2 and IQGAP mediated by BRCA1 and we await further experiments to confirm and define the functionality.

Future research should further explore how truncated PALB2 alters IQGAP1 function in cytoskeletal remodeling and proliferation dynamics, potentially driving tumor cell plasticity in invasive ductal carcinoma. Our combined findings suggest that truncated PALB2 may exert pleiotropic effects on tumor cell behavior by simultaneously modulating genomic maintenance and cytoskeletal organization, probably by moonlighting the roles of these proteins. The functional inhibition or modulation of IQGAP1 in PALB2-deficient contexts, combined with fluorescence resonance energy transfer (FRET) microscopy, must be performed in order to confirm the IQGAP–PALB2 molecular interaction. Transcriptomic profiling may also elucidate the role of IQGAP1 as a molecular switch between proliferation and migration. Clinically, these pathways could represent actionable vulnerabilities; targeting IQGAP1 or its interaction networks [32] might offer novel therapeutic strategies for PALB2-mutated breast cancers, particularly those resistant to conventional DNA repair-targeted treatments [33].

## 5. Conclusions

Our study reported that PALB2 truncation not only compromises homologous recombination but also reprograms cytoskeletal dynamics via altered IQGAP1 expression and localization, thus promoting a migratory and invasive phenotype in IDC. The observed inverse relationship between IQGAP1 and PCNA suggests a regulatory switch between proliferation and invasion. These pleiotropic effects point to the potential of IQGAP1 as both a biomarker and a therapeutic target in PALB2-mutated breast cancers.

## Figures and Tables

**Table 1:** Antibodies used in this study.

Primary Antibodies						
Target		Host/Class		Dilution	Source	Cat.#
PALB2 (N-ter)		Rabbit polyclonal		1:100	Invitrogen ^1^	PA5-48258
PALB2 (C-ter)		Rabbit polyclonal		1:200	Bioss ^2^	BS-0588R
IQGAP1		Rabbit polyclonal		1:500	Millipore ^3^	ABT186
IQGAP1		Mouse monoclonal		1:100	SC Biotechnology ^4^	sc-376021
PCNA		Mouse monoclonal		1:100	Novus ^5^	NB500-106
Β-tubulin		Mouse monoclonal		1:100	SC Biotechnology ^4^	sc-101527
CK7		Rabbit monoclonal		RTU	Biocare Medical ^6^	VLTR339G20
**Secondary Antibodies**						
**Target**	**Conjugation**		**Host/Class**	**Dilution**	**Source**	**Cat.#**
Rabbit IgG	FITC		Goat polyclonal	1:200	Sigma-Aldrich ^7^	F9887
Mouse IgG	DyLight^®^ 650		Goat polyclonal	1:100	Abcam ^8^	ab97018

^1^ Waltham, MA, USA; ^2^ Woburn, MA, USA; ^3^ Bedford, MA, USA; ^4^ Dallas, TX, USA; ^5^ Abingdon, UK; ^6^ Pacheco, CA, USA; ^7^ Irvine, UK; ^8^ Cambridge, UK.

## Data Availability

The data generated in this study are available upon request from the corresponding authors.

## Supplementary Materials

The following supporting information can be downloaded at: https://www.mdpi.com/article/10.3390/biomedicines13081804/s1, Figure S1: PALB2 (green) and IQGAP1 (red) expression in breast cancer control (CC1 and in CC2) mutated breast cancer (P-T1) labelled with antibodies binding to N-terminal or C-terminal domains. Right panels show magnifications of PALB2-IQGAP1 merges.

## Abbreviations

The following abbreviations are used in this manuscript:

IDC	invasive ductal carcinoma
PALB2	partner and localizer of BRCA2
HR	homologous recombination
IQGAP1	IQ motif containing GTPase activating protein 1
P-T	PALB2-truncated variant
CC	breast cancer control
TBS	Tris-buffered saline
LIF	Leica Image Format
PCC	Pearson correlation coefficient
