# Supplementary material for: Functional Disruption of IQGAP1 by Truncated PALB2 in Two Cases of Breast Cancer: Implications for Proliferation and Invasion
Source: Biomedicines. 2025 Jul 23;13(8):1804. doi: 10.3390/biomedicines13081804 (PMC12383994; doi:10.3390/biomedicines13081804)
Supplement: Supplementary file 1 [file biomedicines-13-01804-s001.zip › biomedicines-3709979-supplementary.pdf]

Supplementary Material

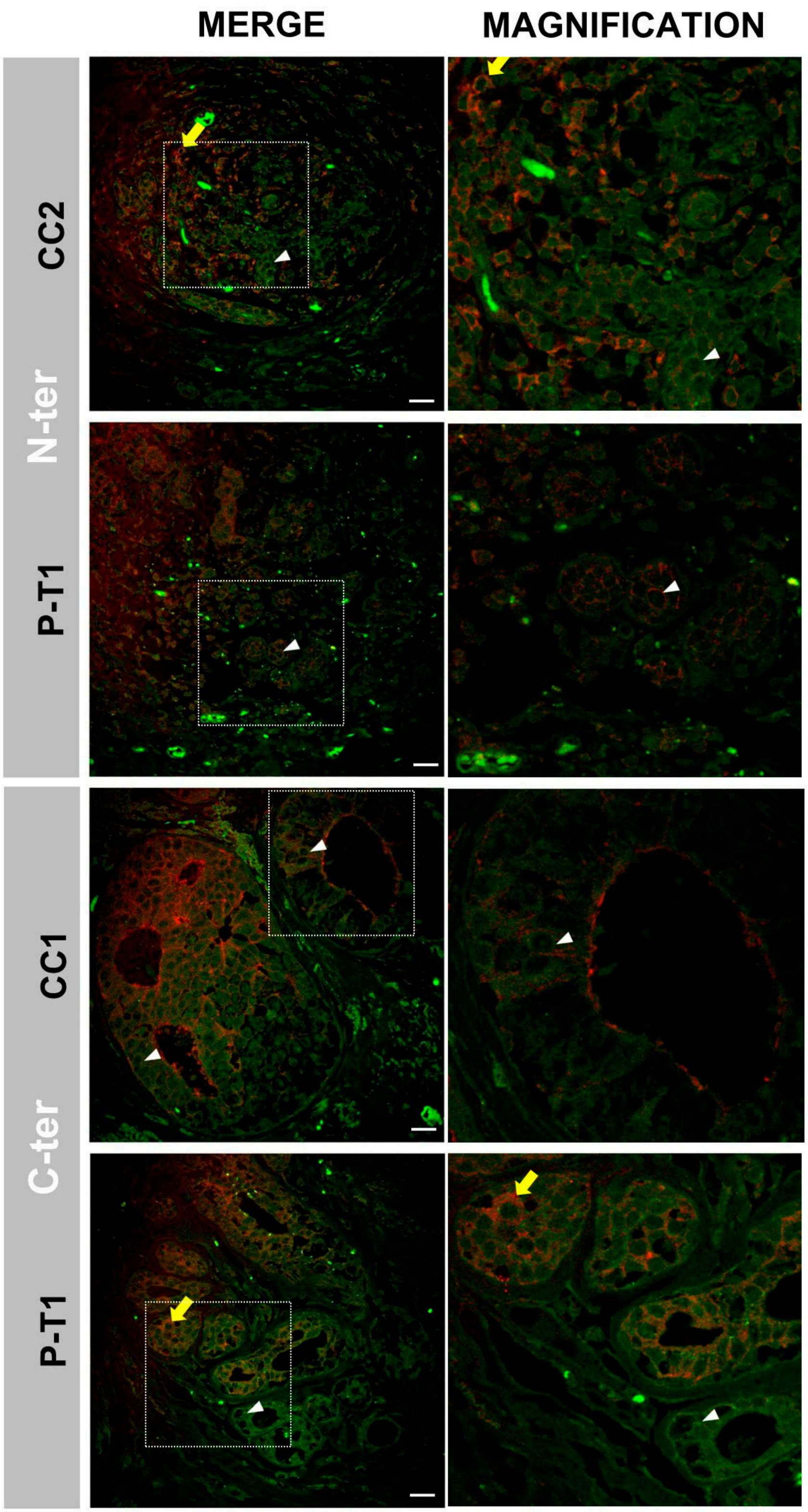

Figure S1. PALB2 (green) and IQGAP1 (red) expression in breast cancer control (CC1 and in CC2) mutated breast cancer (P-T1) labelled with antibodies binding to N-terminal or C-terminal domains. Right panels show magnifications of PALB2-IQGAP1 merges.
